# Predictive Value of FT3/FT4 and Neutrophil‐to‐Lymphocyte Ratios for Post‐Ischemic Stroke Urinary Tract Infection and Their Association With Longitudinal Functional Recovery Trajectories: A Retrospective Cohort Study

**DOI:** 10.1002/brb3.71623

**Published:** 2026-08-04

**Authors:** Zhangyan Geng, Chun Wang, Na Li, Xue Wang, Lihai Zhang, Huiqing Qiu, Ruijing Liang, Xiaohui Jia, Xiaoyu Wang

**Affiliations:** ^1^ Department of Neurology The First Hospital of Hebei Medical University Shijiazhuang China; ^2^ Department of Intensive Care Unit Peking University Third Hospital Qinhuangdao Hospital Qinhuangdao China; ^3^ Department of Geriatrics The First Hospital of Hebei Medical University Shijiazhuang China; ^4^ Department of Respiratory and Critical Care The First Hospital of Hebei Medical University Shijiazhuang China; ^5^ Department of Infectious Diseases The First Hospital of Hebei Medical University Shijiazhuang China

**Keywords:** acute ischemic stroke, FT3/FT4 ratio, generalized estimating equations, longitudinal recovery trajectory, neutrophil‐to‐lymphocyte ratio, urinary tract infection

## Abstract

**Background:**

Urinary tract infection (UTI) is a common complication after acute ischemic stroke (AIS) and is associated with delayed neurological rehabilitation. However, joint biomarkers that reflect stroke‐related neuroendocrine‐immune dysregulation remain insufficiently defined, and the association between UTI and longitudinal functional recovery has not been fully characterized.

**Methods:**

This retrospective cohort study included 122 patients with AIS. Baseline clinical parameters, free triiodothyronine to free thyroxine (FT3/FT4) ratios, and neutrophil‐to‐lymphocyte ratios (NLR) were collected. Multivariate logistic regression was used to identify independent risk factors for UTI. The incremental predictive value of combined biomarkers was evaluated using the area under the receiver operating characteristic curve (AUC), net reclassification improvement (NRI), and integrated discrimination improvement (IDI). A Generalized Estimating Equations (GEE) model was utilized to analyze the longitudinal repeated‐measure modified Rankin Scale (mRS) scores at admission, 28 days, and 180 days.

**Results:**

Among the 122 patients, 33 (27.0%) developed UTI. In the primary multivariable model, a high NLR (≥ 3.78) and a low FT3/FT4 ratio (≤ 0.31) were associated with increased UTI risk. The combined model significantly improved the predictive performance over the baseline clinical model (AUC increased from 0.735 to 0.842, DeLong test *p* = 0.016), with a continuous NRI of 0.635 (*p* < 0.001) and an IDI of 0.188 (*p* < 0.001). Furthermore, the GEE model identified a significant time × group interaction (*p* = 0.014), indicating that UTI was associated with a slower longitudinal functional recovery trajectory. Post‐stroke UTI was associated with poor 180‐day functional outcome in the primary outcome model (adjusted OR = 3.28, 95% CI: 1.15–9.32, *p* = 0.026).

**Conclusions:**

The combination of baseline FT3/FT4 ratio and NLR provides incremental predictive value for post‐ischemic stroke UTI. Furthermore, UTI was associated with a less favorable 6‐month functional recovery trajectory, supporting early risk stratification and prevention‐oriented management.

## Graphical abstract

This graphical abstract illustrates how acute ischemic stroke may be accompanied by neuroendocrine‐immune dysregulation, represented by a low FT3/FT4 ratio and a high neutrophil‐to‐lymphocyte ratio, which together improve early identification of patients at risk for post‐ischemic stroke urinary tract infection. It further shows that urinary tract infection is associated with a less favorable 6‐month functional recovery trajectory and worse long‐term neurological outcomes.

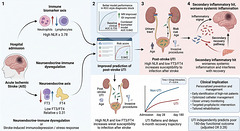

**Abbreviations**
AIS: Acute ischemic stroke
AUC: Area under the curve
BMI: Body mass index
CI: Confidence interval
CRP: C‐reactive protein
EPV: Events per variable
FT3: Free triiodothyronine
FT4: Free thyroxine
GEE: Generalized estimating equations
IDI: Integrated discrimination improvement
IQR: Interquartile range
mRS: modified Rankin Scale
NIHSS: National Institutes of Health Stroke Scale
NLR: Neutrophil‐to‐lymphocyte ratio
NPV: Negative predictive value
NRI: Net reclassification improvement
NTIS: Non‐thyroidal illness syndrome
OR: Odds ratio
PPV: Positive predictive value
SIID: Stroke‐induced immunodepression
UTI: Urinary tract infection
WBC: White blood cell

## 1 Introduction

Acute ischemic stroke (AIS) remains a leading cause of long‐term disability and mortality worldwide, accounts for a substantial proportion of the global disease burden, and imposes considerable socioeconomic costs (Kleindorfer et al. 2021; Feigin et al. 2022). Despite advances in acute reperfusion therapies, including intravenous thrombolysis and mechanical thrombectomy, the post‐stroke rehabilitation phase is frequently complicated by medical morbidities (Dromerick and Edwards 2003). Stroke‐associated infections (SAIs) occur in approximately 30% of patients during the acute and subacute phases, with urinary tract infection (UTI) and pneumonia being the most prevalent (Li et al. 2020). Unlike pneumonia, which often manifests with acute respiratory symptoms, post‐stroke UTI may be clinically subtle but remains associated with fever, prolonged hospitalization, interrupted rehabilitation, and worse functional outcomes (Cheng et al. 2023; Mishra and Hedna 2013).

Importantly, AIS is a heterogeneous disease rather than a single biological entity. Distinct ischemic stroke subtypes, including atherothrombotic or large‐artery infarction, cardioembolic stroke, lacunar infarction, infarction of other determined etiology, and infarction of undetermined etiology, differ in risk‐factor distribution, lesion burden, inflammatory response, stroke severity, and prognosis (Gasull and Arboix 2022). Clinical biomarker studies should therefore acknowledge subtype heterogeneity when interpreting infection risk and recovery outcomes.

Stroke‐induced immunodepression (SIID) and non‐thyroidal illness syndrome (NTIS) are key pathophysiological mechanisms that may predispose patients with AIS to infection (Westendorp et al. 2022; Tai et al. 2023). SIID involves a systemic immune shift characterized by lymphopenia and impaired immune function, which can be clinically approximated by the neutrophil‐to‐lymphocyte ratio (NLR) (Wang et al. 2021; Guo et al. 2024). Concurrently, NTIS is commonly expressed as reduced conversion of thyroxine (T4) to active triiodothyronine (T3), captured by the FT3/FT4 ratio and may reflect the severity of systemic physiological stress (Chen et al. 2025). Although separate associations of NLR and NTIS with stroke outcomes have been reported, their combined value for predicting infectious complications such as UTI has not been sufficiently clarified.

Traditional assessments of post‐stroke functional recovery often rely on static modified Rankin Scale (mRS) evaluations at single time points, such as 90 or 180 days, and therefore may not fully capture the dynamic nature of neurorehabilitation (Seetge et al. 2025; Zhu et al. 2025; Wu et al. 2023). Statistical frameworks such as Generalized Estimating Equations (GEE) can account for repeated‐measure correlations and evaluate whether UTI is associated with a persistently different recovery trajectory over six months (Braakhuis et al. 2021).

Therefore, the primary objective of this retrospective cohort study was to investigate the independent and combined predictive value of baseline FT3/FT4 and NLR for the development of post‐ischemic stroke UTI, using discrimination and reclassification metrics including the area under the curve (AUC), Net Reclassification Improvement (NRI), and Integrated Discrimination Improvement (IDI). The secondary objective was to use a GEE model to characterize longitudinal functional recovery from admission to 28 days and 180 days, while avoiding causal interpretation beyond the limits of the retrospective observational design. This study was conducted and reported in accordance with the STROBE guidelines (von Elm et al. 2007).

## 2 Materials and Methods

### 2.1 Study Population and Design

This was a retrospective, single‐center observational cohort study conducted at a comprehensive tertiary stroke center. We continuously screened patients admitted with a primary diagnosis of acute ischemic stroke (AIS) between January 1, 2024, and January 1, 2025. The study protocol was reviewed and approved by the Institutional Review Board (IRB) of our hospital. Given the retrospective nature of the study, utilizing anonymized and de‐identified electronic medical records, the requirement for written informed consent was formally waived by the ethics committee.

The inclusion criteria were strictly defined as follows: (1) age ≥ 18 years; (2) diagnosis of AIS confirmed by brain computed tomography (CT) or magnetic resonance imaging (MRI) upon admission; (3) time from symptom onset to hospital admission < 24 h; and (4) availability of complete baseline clinical and laboratory data, specifically including a complete blood count with differential and a comprehensive thyroid function panel within 24 h of admission (Chen et al. 2024). To eliminate potential confounding factors that might inherently skew neuroendocrine or immunological profiles, patients meeting any of the following criteria were excluded: (1) clinical or laboratory evidence of active systemic infection (e.g., pneumonia, UTI, or sepsis) present at or preceding the time of admission; (2) history of primary thyroid diseases (hyperthyroidism, hypothyroidism, or thyroid malignancies) or current use of thyroid‐altering medications (e.g., levothyroxine, anti‐thyroid drugs, amiodarone); (3) presence of active autoimmune disorders, hematological malignancies, or ongoing treatment with immunosuppressive agents or systemic corticosteroids; (4) severe hepatic or renal dysfunction (defined as an estimated glomerular filtration rate < 30 mL/min/1.73 m^2^); and (5) loss to follow‐up or incomplete 180‐day functional outcome data (Poisson et al. 2010). Ultimately, a total of 122 eligible AIS patients were included in the final analysis cohort.

### 2.2 Clinical Assessment and Data Collection

Comprehensive baseline demographics, vascular risk factors, and clinical characteristics were systematically extracted from the electronic medical records by two independent trained neurologists blinded to the study's primary hypothesis. Demographic variables included age, gender, and body mass index (BMI). Medical history encompassed documented diagnoses of hypertension, diabetes mellitus, atrial fibrillation/heart disease, current smoking, and alcohol consumption.

Stroke severity upon admission was quantified utilizing the National Institutes of Health Stroke Scale (NIHSS) (Deng et al. 2022). The infarct location was categorized based on neuroimaging findings into large territorial infarcts, brainstem infarcts, multiple infarcts, and others. The presence of consciousness disturbance at admission was meticulously recorded. Furthermore, clinical interventions that represent substantial sources of nosocomial infection risk—defined as “invasive procedures” (e.g., indwelling urinary catheterization, central venous catheter placement, or endotracheal intubation)—and whether the patient required admission to the intensive care unit (ICU) were thoroughly documented.

### 2.3 Laboratory Measurements

Venous blood samples were drawn from the antecubital vein of all enrolled patients within the first 24 h of hospital admission following an overnight fast of at least 8 h. The samples were immediately processed and analyzed by the central laboratory of our institution following standardized automated protocols. The hematological parameters extracted included total white blood cell (WBC) count, neutrophil percentage (*N*%), and absolute neutrophil and lymphocyte counts. The neutrophil‐to‐lymphocyte ratio (NLR) was calculated simply by dividing the absolute neutrophil count by the absolute lymphocyte count.

The neuroendocrine evaluation consisted of thyroid‐stimulating hormone (TSH), free triiodothyronine (FT3), and free thyroxine (FT4), which were measured using electrochemiluminescence immunoassays (Roche Diagnostics). The FT3/FT4 ratio was calculated subsequently as a surrogate marker of peripheral deiodinase activity and non‐thyroidal illness severity. Other inflammatory and metabolic biomarkers, including C‐reactive protein (CRP), D‐dimer, fasting blood glucose, triglycerides, total cholesterol, high‐density lipoprotein (HDL), low‐density lipoprotein (LDL), homocysteine (HCY), uric acid, albumin, prealbumin, and ferritin, were concurrently recorded.

### 2.4 Definition of Post‐Ischemic Stroke Urinary Tract Infection

The primary occurrence of interest during the acute hospitalization phase was the development of post‐ischemic stroke urinary tract infection (UTI). UTI was diagnosed by the treating physicians according to the guidelines established by the Centers for Disease Control and Prevention (CDC) for healthcare‐associated infections. A definitive diagnosis required a combination of newly developed clinical symptoms (e.g., dysuria, urgency, fever > 38.0°C, or suprapubic tenderness) and corroborating laboratory evidence, defined as a positive urinalysis (presence of leukocyte esterase, nitrites, or pyuria [≥ 10 WBC/high‐power field]) or a positive urine culture yielding ≥10^5^ colony‐forming units (CFU)/mL of no more than two species of microorganisms. Only incident UTIs developing at least 48 h after admission were considered stroke‐associated complications.

### 2.5 Longitudinal Functional Outcomes

Functional neurological status was assessed as part of routine clinical follow‐up and retrospectively extracted for the present analysis using the modified Rankin Scale (mRS), a well‐validated ordinal scale ranging from 0 (no symptoms) to 6 (death). To capture the dynamic trajectory of recovery, mRS scores were evaluated at three distinct time points: at baseline (admission), at 28 days post‐stroke, and at 180 days post‐stroke. The evaluations at 28 and 180 days were conducted either during routine outpatient follow‐up clinic visits or via structured telephone interviews by certified stroke research nurses who were blinded to the patients' baseline biomarker profiles and UTI status. For the binary endpoint analysis, a poor functional outcome at 180 days was predefined as an mRS score of ≥ 3, indicating moderate to severe disability or death, whereas an mRS score of 0–2 represented functional independence.

### 2.6 Statistical Analysis

All rigorous statistical analyses were performed using R software (version 4.2.1, R Foundation for Statistical Computing, Vienna, Austria) and SPSS Statistics (version 26.0, IBM Corp., Armonk, NY, USA). The normality of continuous variables was assessed utilizing the Shapiro–Wilk test and visual inspection of Q–Q plots. Normally distributed continuous variables are presented as the mean ± standard deviation (SD) and were compared using the independent samples Student's *t*‐test. Non‐normally distributed continuous variables are reported as the median with interquartile range (IQR) and were compared using the nonparametric Mann–Whitney U test. Categorical variables are expressed as frequencies and percentages (%), and group comparisons were performed using the Pearson Chi‐square (χ^2^) test or Fisher's exact test, as appropriate.

To facilitate clinical applicability, the optimal cut‐off values for continuous biomarkers (NLR and FT3/FT4 ratio) in predicting UTI were determined by calculating the maximum Youden's index (Sensitivity + Specificity – 1) derived from receiver operating characteristic (ROC) curves. Variables exhibiting a marginal trend toward significance (*p* < 0.10) in the univariate analysis were subsequently entered into a multivariate binary logistic regression model using a forward stepwise selection method to identify independent risk factors for post‐ischemic stroke UTI while limiting multicollinearity. Adjusted odds ratios (aOR) and their corresponding 95% confidence intervals (CI) were reported.

To explicitly quantify the incremental predictive value of adding the novel biomarkers (FT3/FT4 and NLR) to a conventional clinical baseline model, we calculated the area under the ROC curve (AUC). The statistical significance of the difference between AUCs was assessed using the DeLong test. Going beyond traditional ROC metrics, we calculated the continuous Net Reclassification Improvement (NRI) and the Integrated Discrimination Improvement (IDI).

For threshold‐based diagnostic characterization requested during peer review, sensitivity, specificity, positive predictive value (PPV), negative predictive value (NPV), and overall diagnostic accuracy were calculated at the model‐specific Youden's index threshold. Wilson score intervals were used for proportions. To examine optimism in the combined prediction model, internal validation was performed with 1000 bootstrap resamples; model optimism was summarized as apparent performance minus test performance, and optimism‐corrected AUC, Brier score, and calibration slope were reported according to prediction‐model reporting principles (Collins et al. 2015).

For the longitudinal analysis of functional recovery, we employed a Generalized Estimating Equations (GEE) model. Because mRS scores were repeatedly measured within the same individuals, a GEE model with an exchangeable working correlation structure was used to account for within‐patient correlation over time. Given that patients with incomplete 180‐day functional outcome data were excluded, the longitudinal analysis should be interpreted as a complete‐case repeated‐measure analysis rather than as a method that fully corrects for missing follow‐up data. Within the GEE framework, we evaluated the Time effect, Group effect (UTI vs. Non‐UTI), and Time × Group interaction. To account for baseline discrepancies in initial stroke severity, the admission NIHSS score was included as an adjustment covariate.

Finally, a secondary multivariable logistic regression model was performed to evaluate the association between UTI and 180‐day poor functional outcome (mRS ≥ 3). A subgroup analysis was additionally conducted across predefined strata, including age ≥ 65 versus < 65 years and infarct location, to evaluate whether the association between UTI and poor outcome appeared directionally similar across clinically relevant groups. Given the limited number of UTI events, subgroup findings were treated as exploratory, and the results were visually represented using a forest plot. Two post hoc sensitivity models were fitted using additional available covariates. For the UTI‐prediction analysis, admission mRS and infarct‐location severity were added to age, invasive procedures, NLR category, and FT3/FT4 category. For the 180‐day outcome analysis, admission mRS and infarct‐location severity were added to the adjusted outcome model. Detailed urinary catheter duration, post‐void residual volume or urinary retention, prestroke disability, TOAST stroke subtype, and reperfusion therapy variables were not available in the retrospective dataset. All statistical tests were two‐tailed, and a *p*‐value of < 0.05 was considered to indicate statistical significance.

## 3 Results

### 3.1 Baseline Characteristics and Clinical Profiles

A total of 122 patients with acute ischemic stroke were included in the final analysis. Among the cohort, 33 patients (27.0%) developed a urinary tract infection (UTI group) during their hospitalization, whereas 89 patients (73.0%) remained free of urinary infections (Non‐UTI group). The baseline demographic, clinical, and laboratory characteristics stratified by the occurrence of UTI are comprehensively detailed in **Table** **1**.

**TABLE 1:** Baseline demographic, clinical, and laboratory characteristics of patients.

Variables	UTI group (*n* = 33)	Non‐UTI group (*n* = 89)	Statistic	*p*‐value
Age (years), mean ± SD	74.2 ± 10.1	67.9 ± 6.9	*t* = 3.125	0.002
Male gender, *n* (%)	16 (48.5%)	51 (57.3%)	χ^2^ = 0.748	0.387
BMI (kg/m^2^), mean ± SD	22.7 ± 3.4	24.1 ± 3.1	*t* = –2.128	0.035
NIHSS score, Median [IQR]	9 [6, 13]	7 [5, 9]	*Z* = –2.610	0.009
Invasive procedures, *n* (%)	11 (33.3%)	12 (13.5%)	χ^2^ = 5.922	0.015
WBC (×10^9^/L), mean ± SD	7.7 ± 2.6	6.4 ± 1.8	*t* = 2.583	0.011
NLR, median [IQR]	4.59 [2.69, 6.84]	2.31 [1.68, 3.40]	*Z* = –4.015	<0.001
FT3 (pmol/L), mean ± SD	3.53 ± 0.65	3.98 ± 0.81	*t* = –2.851	0.005
FT4 (pmol/L), mean ± SD	12.72 ± 1.89	11.58 ± 2.15	*t* = 2.678	0.008
FT3/FT4 ratio, mean ± SD	0.28 ± 0.05	0.35 ± 0.09	*t* = –4.135	<0.001
CRP (mg/L), median [IQR]	15.97 [10.00, 28.40]	12.01 [8.70, 16.36]	*Z* = –2.314	0.021
D‐dimer (mg/L), median [IQR]	1.34 [0.63, 2.70]	0.97 [0.44, 1.67]	*Z* = –2.008	0.045

Abbreviations: BMI, body mass index; CRP, C‐reactive protein; FT3, free triiodothyronine; FT4, free thyroxine; NIHSS, National Institutes of Health Stroke Scale; NLR, neutrophil‐to‐lymphocyte ratio; UTI, urinary tract infection; WBC, white blood cell.

In the univariate analysis, patients who developed a UTI were significantly older than their non‐infected counterparts (74.2 ± 10.1 years vs. 67.9 ± 6.9 years, *p*  = 0.002) and had a slightly lower body mass index (BMI) (22.7 ± 3.4 vs. 24.1 ± 3.1 kg/m^2^, *p*  = 0.035). The UTI group presented with higher neurological deficits at admission, as reflected by higher median NIHSS scores (9 [IQR: 6–13] vs. 7 [IQR: 5–9], *p*  = 0.009). Furthermore, patients in the UTI group were more likely to have undergone invasive procedures, including urinary catheterization (33.3% vs. 13.5%, *p*  = 0.015).

Differences were also observed in the baseline serological biomarker profiles. The UTI group exhibited higher total WBC counts, elevated C‐reactive protein (CRP), and increased D‐dimer levels (all *p* < 0.05).  The median Neutrophil‐to‐Lymphocyte Ratio (NLR) was higher in the UTI group (4.59 [IQR: 2.69–6.84] vs. 2.31 [IQR: 1.68–3.40], *p* < 0.001). Conversely, concerning the neuroendocrine axis, patients complicated with UTI presented with significantly lower serum free T3 levels (3.53 ± 0.65 vs. 3.98 ± 0.81 pmol/L, *p* = 0.005) and a lower FT3/FT4 ratio (0.28 ± 0.05 vs. 0.35 ± 0.09, *p* < 0.001). There were no statistically significant differences regarding gender distribution, smoking or alcohol habits, and the prevalence of comorbidities such as hypertension, diabetes, or pre‐existing heart disease between the two cohorts (all *p* > 0.05).

### 3.2 Primary and Sensitivity Multivariable Analyses for Post‐Ischemic Stroke UTI

To identify the independent risk factors for the development of post‐stroke UTI, variables demonstrating a *p*‐value < 0.10 in the univariate analysis were integrated into a multivariate stepwise logistic regression model. Based on the ROC curve analysis for predicting UTI, the continuous variables NLR and FT3/FT4 ratio were dichotomized using optimal cut‐off values of 3.78 and 0.31, respectively.

Candidate variables entered into the multivariable UTI‐prediction model included age, invasive procedures, NIHSS score, BMI, CRP, D‐dimer, NLR category, and FT3/FT4 category. After forward stepwise selection, the final model retained age, invasive procedures, NLR ≥ 3.78, and FT3/FT4 ratio ≤ 0.31. As presented in **Table** **2**, a high baseline NLR (≥ 3.78) independently increased the risk of UTI (aOR = 3.61, 95% CI: 1.34–9.74, *p* = 0.011). Similarly, a low FT3/FT4 ratio (≤ 0.31) was an independent neuroendocrine predictor (aOR = 4.25, 95% CI: 1.58–11.45, *p* = 0.004). Advanced age (aOR = 1.08 per year increase, *p* = 0.006) and the requirement for invasive procedures (aOR = 3.15, *p* = 0.038) also remained in the final model.

**TABLE 2:** Multivariate logistic regression analysis for independent risk factors of post‐ischemic stroke UTI.

Variables	β	SE	Wald χ^2^	*p*‐value	Adjusted OR (95% CI)
Age (continuous)	0.081	0.030	7.458	0.006	1.08 (1.02–1.15)
Invasive procedures (yes vs. no)	1.147	0.552	4.321	0.038	3.15 (1.07–9.29)
NLR (≥ 3.78 vs. < 3.78)	1.284	0.506	6.425	0.011	3.61 (1.34–9.74)
FT3/FT4 ratio (≤ 0.31 vs. > 0.31)	1.447	0.505	8.196	0.004	4.25 (1.58–11.45)
Constant	−8.665	2.511	11.905	0.001	0.000

*Note*: Candidate variables included age, invasive procedures, NIHSS score, BMI, CRP, D‐dimer, NLR category, and FT3/FT4 category. Only variables retained in the final stepwise model are shown.

In the post hoc UTI‐prediction sensitivity model additionally incorporating admission mRS and infarct‐location severity, the associations for high NLR (aOR = 2.28, 95% CI: 0.75–6.93, *p* = 0.146) and low FT3/FT4 ratio (aOR = 2.28, 95% CI: 0.73–7.11, *p* = 0.157) remained directionally consistent but were no longer statistically significant. Admission mRS was associated with UTI risk in this exploratory model (aOR = 1.62, 95% CI: 1.02–2.58, *p* = 0.040). These sensitivity results should be interpreted cautiously because of the limited number of UTI events.

### 3.3 Incremental Predictive Value and Reclassification (NRI and IDI)

**FIGURE 1:**
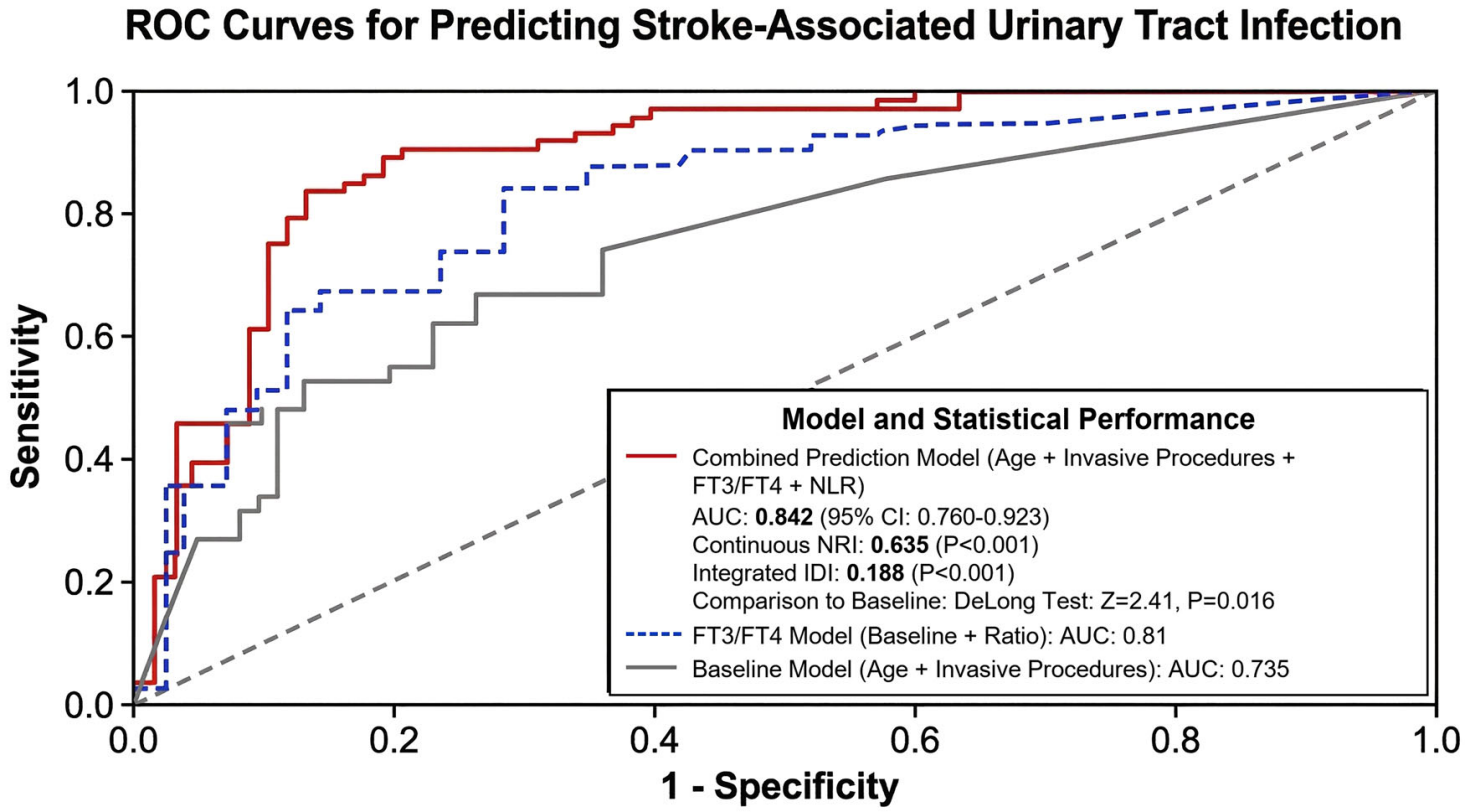
Receiver operating characteristic (ROC) curves for predicting post‐ischemic stroke urinary tract infection (UTI). The baseline model (gray solid line) is constructed based on traditional clinical risk factors, comprising age and the requirement for invasive procedures. The single‐biomarker model (blue dashed line) illustrates the addition of the serum FT3/FT4 ratio to the baseline model. The combined model (red thick line) incorporates both the FT3/FT4 ratio and the neutrophil‐to‐lymphocyte ratio (NLR) into the baseline model. For visual clarity, the figure displays the baseline model, the FT3/FT4 single‐biomarker model, and the combined model, whereas the full four‐model comparison is summarized in **Table** **3**. The combined model demonstrated a larger area under the curve (AUC = 0.842) than the baseline model (AUC = 0.735, DeLong test *p* = 0.016), indicating improved discriminative performance. Additional threshold‐based diagnostic metrics are summarized in **Table** **4**.

To assess whether the neuroendocrine‐immune biomarker combination provided additive prognostic utility beyond standard clinical assessment, we established a baseline clinical model comprising age and invasive procedures. We then evaluated the incremental value of adding NLR, the FT3/FT4 ratio, and both biomarkers combined (**Table** **3** and **Figure** **1**).

**TABLE 3:** Incremental predictive value of FT3/FT4 and NLR over the baseline clinical model.

Models	AUC (95% CI)	*p* vs. baseline	NRI (95% CI)	IDI (95% CI)
Baseline model (age + invasive procedures)	0.735 (0.632–0.838)	Reference	Ref.	Ref.
Baseline + NLR	0.796 (0.697–0.895)	0.085	0.412 (0.125–0.698)*	0.105 (0.022–0.188)*
Baseline + FT3/FT4 ratio	0.812 (0.720–0.904)	0.042	0.485 (0.188–0.781)*	0.128 (0.045–0.211)**
Combined model (baseline + NLR + FT3/FT4)	0.842 (0.760–0.923)	0.016	0.635 (0.342–0.928)**	0.188 (0.085–0.291)**

* *p* < 0.05, ^**^ *p* < 0.001 for NRI/IDI analyses vs. baseline model.

The baseline clinical model yielded an AUC of 0.735 (95% CI: 0.632–0.838). The addition of either the NLR or the FT3/FT4 ratio alone increased the AUC to 0.796 and 0.812, respectively. The combined model (Baseline + NLR + FT3/FT4 ratio) achieved the highest discriminatory power with an AUC of 0.842 (95% CI: 0.760–0.923). The DeLong test confirmed that the AUC of the combined model was significantly superior to that of the baseline model (*Z*  = 2.41, *p*  = 0.016).

Beyond traditional ROC analysis, the clinical utility was further evaluated utilizing reclassification metrics. The incorporation of both the NLR and the FT3/FT4 ratio into the baseline model resulted in a continuous Net Reclassification Improvement (NRI) of 0.635 (95% CI: 0.342–0.928, *p* < 0.001) and an Integrated Discrimination Improvement (IDI) of 0.188 (95% CI: 0.085–0.291, *p* < 0.001). These parameters indicate improved risk stratification when using the combined model compared with the baseline model alone.

For the additional threshold‐based diagnostic performance analysis, the combined model showed a sensitivity of 84.4% (95% CI: 68.2%–93.1%), specificity of 74.4% (95% CI: 64.6%–82.3%), PPV of 54.0% (95% CI: 40.4%–67.0%), NPV of 93.1% (95% CI: 84.8%–97.0%), and overall accuracy of 77.0% (95% CI: 68.8%–83.6%) (**Table** **4**). Internal validation with 1000 bootstrap resamples indicated modest optimism in discrimination: the apparent AUC derived from the bootstrap validation procedure was 0.815, mean optimism was 0.026, and the optimism‐corrected AUC was 0.790. The optimism‐corrected Brier score was 0.149, and the optimism‐corrected calibration slope was 0.873. The prespecified ROC comparison in **Table** **3** and the bootstrap‐derived apparent and optimism‐corrected indices are therefore reported separately. At the model‐specific Youden's index threshold, the combined model increased sensitivity and NPV compared with the baseline clinical model, whereas specificity, PPV, and overall accuracy were lower. Therefore, the combined biomarker model should be interpreted primarily as improving discrimination and reclassification and as a potential screening‐oriented risk‐stratification tool, rather than as a standalone diagnostic rule.

**TABLE 4:** Threshold‐based diagnostic performance of multivariable prediction models and bootstrap internal validation of the combined model for post‐ischemic stroke UTI.

Model	Sensitivity (95% CI)	Specificity (95% CI)	PPV (95% CI)	NPV (95% CI)	Accuracy (95% CI)
Baseline clinical model	65.6% (48.3–79.6)	90.0% (82.1–94.6)	70.0% (52.1–83.3)	88.0% (79.8–93.2)	83.6% (76.0–89.1)
Baseline + NLR	81.2% (64.7–91.1)	77.8% (68.2–85.1)	56.5% (42.2–69.8)	92.1% (83.8–96.3)	78.7% (70.6–85.0)
Baseline + FT3/FT4 ratio	68.8% (51.4–82.0)	83.3% (74.3–89.6)	59.5% (43.5–73.7)	88.2% (79.7–93.5)	79.5% (71.5–85.7)
Combined model	84.4% (68.2–93.1)	74.4% (64.6–82.3)	54.0% (40.4–67.0)	93.1% (84.8–97.0)	77.0% (68.8–83.6)

*Note*: Diagnostic performance was calculated at the model‐specific Youden's index threshold. Bootstrap internal validation was performed for the combined model only; it used 1000 resamples and yielded mean optimism of 0.026, optimism‐corrected AUC of 0.790, optimism‐corrected Brier score of 0.149, and optimism‐corrected calibration slope of 0.873.

Abbreviations: PPV, positive predictive value; NPV, negative predictive value; CI, confidence interval.

### 3.4 Association Between UTI and Longitudinal Functional Recovery Trajectories

**FIGURE 2:**
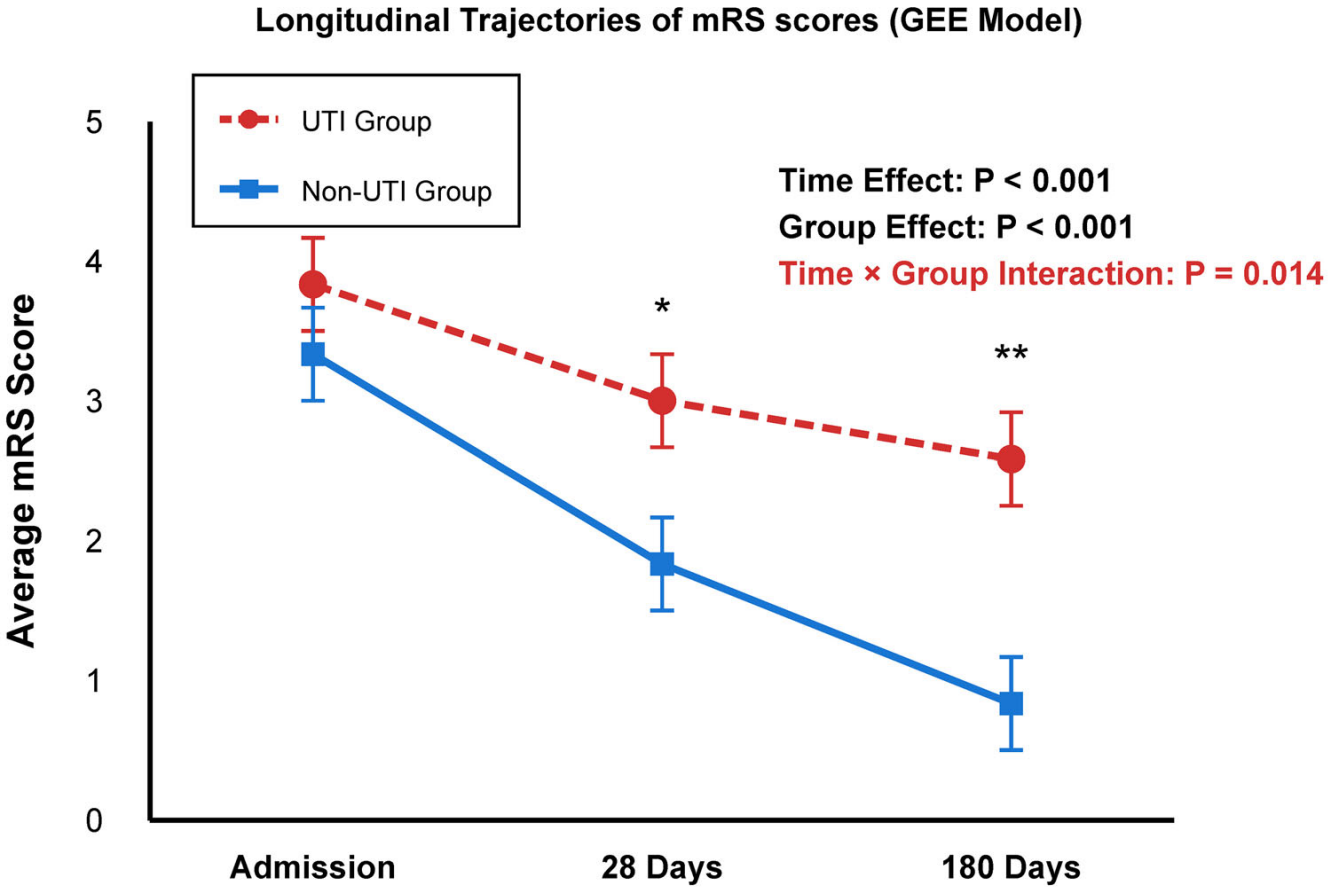
Longitudinal functional recovery trajectories based on the Generalized Estimating Equations (GEE) model. The line graphs depict the dynamic evolution of the mean modified Rankin Scale (mRS) scores at admission, 28 days, and 180 days post‐stroke. The red dashed line represents the UTI group, and the blue solid line represents the Non‐UTI group. Higher mRS scores indicate more severe neurological functional deficits. Error bars indicate the standard error of the mean (SEM). The GEE analysis revealed significant main effects for both Time (*p* < 0.001) and Group (*p* < 0.001), and a significant Time × Group interaction (*p* = 0.014), indicating that UTI was associated with a less favorable recovery slope over time.

To capture how post‐stroke UTI was associated with the recovery process over a half‐year period, we analyzed the repeated‐measure mRS scores at admission, 28 days, and 180 days using a Generalized Estimating Equations (GEE) model. The longitudinal trajectories are visually depicted in **Figure** **2**.

The GEE analysis, adjusted for baseline NIHSS scores, yielded the following results. A significant Time main effect was observed (Wald χ^2^ = 125.4, *p* < 0.001), showing a general trend of decreasing mRS scores from admission to 180 days across the entire cohort. A significant Group main effect was also observed (Wald χ^2^ = 14.2, *p* < 0.001), with the UTI group demonstrating higher mean mRS scores compared to the non‐UTI group across the assessed time points.

Furthermore, the GEE model identified a significant Time × Group interaction (Wald χ^2^ = 8.56, *p* = 0.014). As shown in **Figure** **2**, the interaction indicates a difference in the slopes of functional change between the two cohorts. Specifically, the rate of decline in mRS scores over the 180‐day period was less pronounced in the UTI group compared with the non‐UTI group, resulting in a widening difference in mean mRS scores at the 28‐day and 180‐day follow‐up points relative to admission.

### 3.5 Multivariable and Subgroup Analyses for 180‐Day Poor Functional Outcome

Finally, we evaluated the determinants of long‐term poor functional outcome, defined as an mRS score of ≥ 3 at 180 days. Candidate variables for the 180‐day outcome model included baseline NIHSS score, age category, UTI status, FT3/FT4 category, gender, BMI, hypertension, and diabetes. After variable selection, baseline NIHSS score, age ≥ 65 years, UTI status, and FT3/FT4 ratio ≤ 0.31 were retained in the final model (**Table** **5**). Post‐stroke UTI was associated with poor 180‐day outcome (adjusted OR = 3.28, 95% CI: 1.15–9.32, *p* = 0.026). Concurrently, an initially low FT3/FT4 ratio (≤ 0.31) also predicted poor long‐term recovery (adjusted OR = 2.65, 95% CI: 1.04–6.76, *p* = 0.041).

**TABLE 5:** Final multivariable logistic regression model for 180‐day poor functional outcome (mRS ≥ 3).

Variables	β	SE	Wald χ^2^	*p*‐value	Adjusted OR (95% CI)
Baseline NIHSS score	0.215	0.082	6.875	0.009	1.24 (1.05–1.46)
Age (≥ 65 vs. < 65)	1.182	0.595	3.945	0.047	3.26 (1.01–10.47)
Complicated with UTI (yes vs. no)	1.188	0.534	4.952	0.026	3.28 (1.15–9.32)
FT3/FT4 ratio (≤ 0.31 vs. > 0.31)	0.975	0.478	4.161	0.041	2.65 (1.04–6.76)

*Note*: Candidate variables included baseline NIHSS score, age category, UTI status, FT3/FT4 category, gender, BMI, hypertension, and diabetes. Only variables retained in the final stepwise model are displayed.

In a post hoc sensitivity model additionally incorporating admission mRS and infarct‐location severity, the association between UTI and 180‐day poor functional outcome remained directionally consistent but was not statistically significant (aOR = 2.30, 95% CI: 0.40–13.15, *p* = 0.350). The FT3/FT4 ratio also showed a consistent association with poor outcome (aOR = 5.61, 95% CI: 1.01–31.11, *p* = 0.048). These findings should be interpreted cautiously because the model remained constrained by the limited number of outcome events.

**FIGURE 3:**
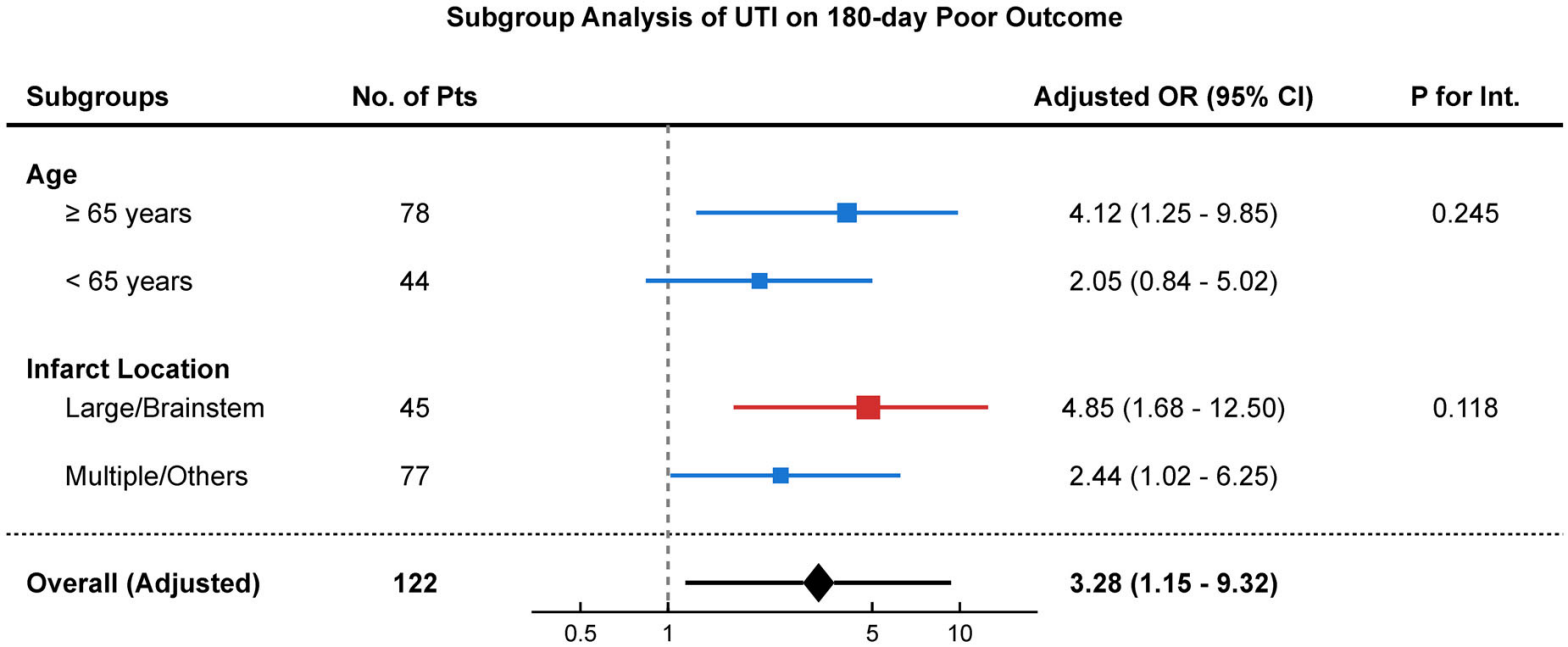
Forest plot of exploratory subgroup analyses evaluating the association between UTI and 180‐day poor functional outcome (mRS ≥ 3). Subgroups were predefined based on key clinical characteristics, including age stratification (≥ 65 vs. < 65 years) and infarct location (large territorial or brainstem infarcts vs. multiple or other locations). Multivariate logistic regression models adjusted for gender, BMI, hypertension, and diabetes were used to calculate adjusted odds ratios (aORs) and 95% confidence intervals (CIs). The black central diamond represents the overall adjusted aOR, the colored squares represent subgroup‐specific aORs, and horizontal lines denote 95% CIs. Because the number of UTI events was limited, these subgroup findings should be interpreted cautiously and considered hypothesis‐generating.

Because only 33 UTI cases were observed, the subgroup analyses were interpreted cautiously and descriptively (**Figure** **3**). The forest plot showed that the point estimates for UTI generally favored worse outcomes across predefined subgroups; however, confidence intervals were wide, and none of the interaction tests reached statistical significance (all *p* for interaction > 0.05). These exploratory findings should therefore be viewed as hypothesis‐generating rather than confirmatory evidence of subgroup consistency.

## 4 Discussion

In this retrospective cohort study, we investigated the interplay between baseline neuroendocrine‐immune biomarkers and the development of post‐stroke UTI, and further characterized the association between UTI and longitudinal neurological recovery. Our study yielded three main findings. First, in the primary multivariable model, a diminished FT3/FT4 ratio and an elevated NLR at admission were associated with post‐stroke UTI, although these associations were attenuated after additional adjustment for admission mRS and infarct‐location severity in the post hoc sensitivity model; the integration of these two biomarkers improved predictive performance beyond traditional clinical variables, as reflected by NRI and IDI. Second, the GEE model described different longitudinal functional recovery trajectories between the UTI and non‐UTI groups; UTI was associated with a less favorable 6‐month recovery slope, but causality cannot be inferred from the retrospective design. Third, in the primary outcome model, UTI and a low FT3/FT4 ratio were associated with 180‐day poor functional outcomes; however, after additional adjustment for admission mRS and infarct‐location severity, the association between UTI and poor outcome was directionally consistent but no longer statistically significant, whereas the FT3/FT4 ratio remained associated with poor outcome. Subgroup analyses were exploratory and underpowered.

Susceptibility to infection after acute ischemic stroke is closely related to stroke‐induced immunodepression (SIID) (Westendorp et al. 2022). Following acute cerebral ischemia, the central nervous system may downregulate systemic immunity through activation of the hypothalamic–pituitary–adrenal (HPA) axis and sympathetic nervous system as a counter‐regulatory response to neuroinflammation (Westendorp et al. 2022). This response may be accompanied by systemic lymphopenia and splenic atrophy (Wang et al. 2021), impaired phagocytic functions (Wang et al. 2023), and a period of vulnerability to bacterial infection (Westendorp et al. 2022). The NLR is an accessible hematological marker that captures the balance between innate inflammatory activation and adaptive immune suppression (Wang et al. 2021). In our cohort, the optimal NLR cut‐off for predicting post‐stroke UTI was 3.78. This threshold is broadly consistent with prior reports, although cut‐offs vary according to the infection type and outcome studied. For example, studies of stroke‐associated infection have reported optimal NLR cut‐offs of approximately 4.7 for post‐stroke pneumonia (Gens et al. 2021) and 5.79 for overall post‐stroke infections (He et al. 2020). The lower cut‐off in our study may reflect the more insidious clinical course of UTI compared with pneumonia, while also emphasizing that NLR is a non‐specific inflammatory marker. Combining NLR with neuroendocrine markers may therefore improve risk characterization.

The neuroendocrine network may also shift toward non‐thyroidal illness syndrome (NTIS), typically characterized by low serum FT3 levels with normal or decreased TSH and FT4 levels (Yu et al. 2024; Taroza et al. 2020). After stroke, the peripheral conversion of T4 to metabolically active T3 by deiodinases may be reduced (Taroza et al. 2020). A low FT3/FT4 ratio may therefore reflect systemic stress and altered thyroid hormone metabolism. Thyroid hormones also participate in innate immune responses, macrophage polarization, and pathogen clearance (Rubingh et al. 2020). In our cohort, an FT3/FT4 ratio ≤ 0.31 was associated with increased UTI risk. The observed NRI and IDI values suggest that evaluating the immune component represented by NLR together with the neuroendocrine component represented by FT3/FT4 can provide complementary information beyond conventional clinical parameters (Han et al. 2025). Recent clinical studies similarly support the relevance of combining metabolic and inflammatory markers, showing that a lower T3/T4 ratio is correlated with inflammatory indices, including SII and NLR, and is associated with mortality in stroke survivors (Zhang et al. 2024). Although initial stroke severity measured by NIHSS is an established clinical determinant, it was excluded from the baseline prediction model during stepwise selection, probably because of overlap with downstream stress‐related inflammatory and endocrine responses.

More broadly, our study fits within a growing body of clinical stroke research examining neuroendocrine‐immune crosstalk. Beyond NLR and FT3/FT4, other investigations have used systemic stress indicators such as cortisol in combination with inflammatory indices. Acute ischemia can activate the HPA axis and increase cortisol secretion, and recent studies have reported that elevated cortisol and higher NLR are independently and positively correlated with greater stroke severity in patients with AIS (Mohd Azmi et al. 2021; Amalia et al. 2023). We focused on FT3/FT4 combined with NLR because these markers are routinely collected, inexpensive, and may provide a practical clinical window into metabolic regulation and immune balance.

A further contribution of the present study is the application of GEE methodology to model longitudinal functional recovery trajectories. Traditional stroke outcome research often relies on static, dichotomized mRS outcomes at a single time point, such as 90 or 180 days, whereas the GEE approach captures the dynamic nature of rehabilitation and allows comparison of recovery slopes between groups (Fugl‐Meyer et al. 1975). The trajectory plot (**Figure** **2**) shows that both groups improved over time, but the UTI group showed a less favorable recovery slope. This association is biologically plausible because systemic infection and inflammation may increase circulating cytokines such as IL‐6 and TNF‐α, potentially aggravating neuroinflammatory responses and impairing neuroplasticity. However, these mechanistic explanations remain inferential in the present retrospective observational study and should be tested in prospective studies with serial inflammatory measurements.

The clinical implications of our findings should be interpreted as risk‐stratification support rather than proof that biomarker‐guided intervention changes outcomes. Baseline FT3/FT4 and NLR may help identify patients who warrant closer UTI surveillance and careful catheter‐related management, particularly because previous stroke cohorts have linked post‐stroke UTI or post‐stroke infection to age, stroke severity, catheter‐related mechanisms, and inflammatory markers (Li et al. 2020; Poisson et al. 2010; Gens et al. 2021; He et al. 2020; Jitpratoom and Boonyasiri 2023). Potential preventive measures include avoiding unnecessary indwelling urinary catheters, removing catheters as early as clinically feasible, monitoring urinary symptoms and urinalysis when indicated, and considering intermittent catheterization protocols in appropriate patients, although the effectiveness of biomarker‐guided catheter‐management strategies requires prospective evaluation. Because UTI was associated with less favorable long‐term functional recovery, affected patients may also require careful rehabilitation planning and follow‐up (Knecht et al. 2021). Future clinical trials should investigate whether targeted prevention strategies in patients with high‐risk biomarker profiles can reduce UTI incidence and improve functional recovery.

We acknowledge several limitations. First, the retrospective, single‐center design may introduce selection and referral biases, and the modest sample size (*n* = 122) limited the number of covariates that could be included in multivariable models. The primary combined model included approximately eight UTI events per variable, indicating a risk of overfitting; therefore, we added bootstrap internal validation and reported optimism‐corrected performance. Second, several clinically important predictors of post‐stroke UTI were unavailable in the retrospective dataset, including detailed duration of urinary catheterization, post‐void residual volume or urinary retention, prestroke disability, TOAST stroke subtype, and reperfusion therapy status. We conducted an exploratory sensitivity model using the additional available variables of admission mRS and infarct‐location severity, but prospective datasets should collect these variables systematically. Third, NLR and FT3/FT4 were evaluated using a single baseline measurement at admission; dynamic changes in these markers and their temporal relationship with UTI onset were not captured. Fourth, detailed microbiological data on uropathogens and antimicrobial resistance were not incorporated. Fifth, subgroup analyses were underpowered because only 33 UTI events occurred, and they should be considered hypothesis‐generating. Lastly, external validation in independent, larger, multicenter prospective cohorts is essential before the combined prediction model can be implemented in routine clinical stroke pathways.

A future line of research should specifically evaluate whether lacunar and non‐lacunar ischemic strokes differ in the predictive value of FT3/FT4 and NLR for post‐stroke UTI. Lacunar infarcts have distinct pathophysiology, clinical features, and long‐term prognosis compared with other ischemic cerebrovascular diseases (Arboix et al. 2010). Prospective cohorts with standardized TOAST classification, lesion‐volume quantification, and serial biomarker sampling are needed to determine whether stroke subtype modifies biomarker‐infection associations.

Other future directions include external validation of the combined biomarker model, prospective assessment of urinary catheter duration and post‐void residual volume, integration of microbiological profiles and antimicrobial resistance patterns, evaluation of biomarker kinetics before and after infection onset, and testing whether biomarker‐guided UTI surveillance or catheter‐management protocols can improve functional recovery.

## 5 Conclusions

In conclusion, baseline FT3/FT4 ratio and NLR were associated with post‐ischemic stroke UTI and improved model discrimination in this single‐center retrospective cohort. In a longitudinal GEE framework, post‐stroke UTI was associated with a slower and less favorable 6‐month functional recovery trajectory, although causal inference cannot be established from this retrospective study. Routine baseline screening of these accessible biomarkers may support individualized infection surveillance and catheter‐management strategies in high‐risk ischemic stroke patients, pending prospective external validation.

## Author Contributions

**Xue Wang**: investigation, methodology, formal analysis, data curation. **Chun Wang**: methodology, investigation, formal analysis, data curation, writing – original draft. **Lihai Zhang**: methodology, formal analysis, data curation. **Xiaohui Jia**: formal analysis, methodology. **Na Li**: investigation, methodology, formal analysis, data curation. **Xiaoyu Wang**: conceptualization, investigation, writing – review and editing, writing – original draft, formal analysis, methodology, supervision, resources. **Ruijing Liang**: methodology, investigation, formal analysis. **Zhangyan Geng**: conceptualization, investigation, methodology, formal analysis, data curation, writing – original draft. **Huiqing Qiu**: formal analysis, methodology, data curation.

## Funding

This study was supported by the Medical Science Research Project of Hebei (Grant No. 20241539). The funder had no role in the conceptualization, design, data collection, analysis, decision to publish, or preparation of the manuscript.

## Ethics Statement

This study was reviewed and approved by the Institutional Review Board (IRB) of The First Hospital of Hebei Medical University (Approval No. 2023S00459). Given the retrospective nature of the study using anonymized electronic medical records, the requirement for written informed consent was formally waived by the ethics committee.

## Conflicts of Interest

The authors declare that they have no competing interests.

## Data Availability

The datasets used and/or analyzed during the current study are available from the corresponding author on reasonable request.

## References

[brb371623-bib-0001] Amalia, L. , M. D. Garyani , and N. Lailiyya . 2023. “Increasing of Cortisol Level and Neutrophil‐Lymphocyte‐Ratio Are Associated With Severity Level and Sleep Disturbances in Acute Ischemic Stroke.” International Journal of General Medicine 16: 5439–5448. 10.2147/IJGM.S439149. 38021057 PMC10676643

[brb371623-bib-0002] Arboix, A. , J. Massons , L. García‐Eroles , et al. 2010. “Nineteen‐Year Trends in Risk Factors, Clinical Characteristics and Prognosis in Lacunar Infarcts.” Neuroepidemiology 35, no. 3: 231–236. 10.1159/000319460. 20861654

[brb371623-bib-0003] Braakhuis, H. E. M. , M. A. M. Berger , R. G. R. H. Regterschot , et al. 2021. “Physical Activity Dimensions After Stroke: Patterns and Relation With Lower Limb Motor Function.” Journal of NeuroEngineering and Rehabilitation 18, no. 1: 171. 10.1186/s12984-021-00960-x. 34895265 PMC8666008

[brb371623-bib-0004] Chen, B. , L. Xu , L. Guo , J. Hou , and F. Han . 2025. “Efficacy of Preoperative Free Thyroxine/Free Triiodothyronine Ratio in Evaluating Prognosis After Radiofrequency Ablation for Persistent Atrial Fibrillation.” Journal of Qingdao University (Medical Sciences) 61, no. 3: 431–435.

[brb371623-bib-0005] Chen, L. , L. Zhang , Y. Li , Q. Zhang , Q. Fang , and X. Tang . 2024. “Association of the Neutrophil‐to‐Lymphocyte Ratio With 90‐Day Functional Outcomes in Patients With Acute Ischemic Stroke.” Brain Sciences 14, no. 3: 250. 10.3390/brainsci14030250. 38539638 PMC10968739

[brb371623-bib-0006] Cheng, S. , J. Li , J. Guo , et al. 2023. “Effect of Post‐Stroke Infection on Discharge Outcomes in Patients With Acute Ischemic Stroke.” Chinese Journal of Stroke 18, no. 8: 885–890.

[brb371623-bib-0007] Collins, G. S. , J. B. Reitsma , D. G. Altman , and K. G. M. Moons . 2015. “Transparent Reporting of a Multivariable Prediction Model for Individual Prognosis or Diagnosis (TRIPOD): The TRIPOD Statement.” Annals of Internal Medicine 162, no. 1: 55–63. 10.7326/M14-0697. 25560714

[brb371623-bib-0008] Deng, P. P. , N. Wu , X. J. Chen , F. L. Chen , H. S. Xu , and G. S. Bao . 2022. “NIHSS—The Alberta Stroke Program Early CT Score Mismatch in Guiding Thrombolysis in Patients With Acute Ischemic Stroke.” Journal of Neurology 269, no. 3: 1515–1521. 10.1007/s00415-021-10704-5. 34318373 PMC8315493

[brb371623-bib-0009] Dromerick, A. W. , and D. F. Edwards . 2003. “Relation of Postvoid Residual to Urinary Tract Infection During Stroke Rehabilitation.” Archives of Physical Medicine and Rehabilitation 84, no. 9: 1369–1372. 10.1016/S0003-9993(03)00201-6. 13680576

[brb371623-bib-0010] Feigin, V. L. , M. Brainin , B. Norrving , et al. 2022. “World Stroke Organization (WSO): Global Stroke Fact Sheet 2022.” International Journal of Stroke 17, no. 1: 18–29. 10.1177/17474930211065917. 34986727

[brb371623-bib-0011] Fugl‐Meyer, A. R. , L. Jääskö , I. Leyman , S. Olsson , and S. Steglind . 1975. “The Post‐Stroke Hemiplegic Patient. 1. A Method for Evaluation of Physical Performance.” Journal of Rehabilitation Medicine 7, no. 1: 13–31. 10.2340/1650197771331. 1135616

[brb371623-bib-0012] Gasull, T. , and A. Arboix . 2022. “Molecular Mechanisms and Pathophysiology of Acute Stroke: Emphasis on Biomarkers in the Different Stroke Subtypes.” International Journal of Molecular Sciences 23, no. 16: 9476. 10.3390/ijms23169476. 36012743 PMC9409332

[brb371623-bib-0013] Gens, R. , A. Ourtani , A. De Vos , J. De Keyser , and S. De Raedt . 2021. “Usefulness of the Neutrophil‐to‐Lymphocyte Ratio as a Predictor of Pneumonia and Urinary Tract Infection Within the First Week After Acute Ischemic Stroke.” Frontiers in Neurology 12: 671739. 10.3389/fneur.2021.671739. 34054712 PMC8155535

[brb371623-bib-0014] Guo, J. , D. Wang , J. Jia , et al. 2024. “Neutrophil‐to‐Lymphocyte Ratio, Lymphocyte‐to‐Monocyte Ratio and Platelet‐to‐Lymphocyte Ratio as Predictors of Short‐ and Long‐Term Outcomes in Ischemic Stroke Patients With Atrial Fibrillation.” Journal of Inflammation Research 17: 6661–6672. 10.2147/JIR.S480513. 39345895 PMC11430226

[brb371623-bib-0015] Han, C. , L.e Wang , C. Liu , et al. 2025. “FT3/FT4 Enhances Risk Assessment in Patients with Non‐ST‐Segment Elevation Acute Coronary Syndrome Undergoing Percutaneous Coronary Intervention Based on GRACE 2.0 Score.” Angiology 76, no. 2: 125–140. 10.1177/00033197231199228. 37876209

[brb371623-bib-0016] He, L. , J. Wang , F. Wang , L. Zhang , L. Zhang , and W. Zhao . 2020. “Increased Neutrophil‐to‐Lymphocyte Ratio Predicts the Development of Post‐Stroke Infections in Patients With Acute Ischemic Stroke.” BMC Neurology 20, no. 1: 328. 10.1186/s12883-020-01914-x. 32873248 PMC7460775

[brb371623-bib-0017] Jitpratoom, P. , and A. Boonyasiri . 2023. “Determinants of Urinary Tract Infection in Hospitalized Patients With Acute Ischemic Stroke.” BMC Neurology 23, no. 1: 251. 10.1186/s12883-023-03296-2. 37391711 PMC10311730

[brb371623-bib-0018] Kleindorfer, D. O. , A. Towfighi , S. Chaturvedi , et al. 2021. “2021 Guideline for the Prevention of Stroke in Patients with Stroke and Transient Ischemic Attack: A Guideline from the American Heart Association/American Stroke Association.” Stroke; A Journal of Cerebral Circulation 52, no. 7: e364–e467. 10.1161/STR.0000000000000375. 34024117

[brb371623-bib-0019] Knecht, S. , V. K. Jalali , T. Schmidt‐Wilcke , and B. Studer . 2021. “Krankenhausmedizinische Interventionen in der Neurologischen Anschlussrehabilitation.” Der Nervenarzt 92, no. 2: 137–143. 10.1007/s00115-020-01021-9. 33125513 PMC7597745

[brb371623-bib-0020] Li, Y. M. , J. H. Xu , and Y. X. Zhao . 2020. “Predictors of Urinary Tract Infection in Acute Stroke Patients: A Cohort Study.” Medicine 99, no. 27: e20952. 10.1097/MD.0000000000020952. 32629702 PMC7337551

[brb371623-bib-0021] Mishra, M. , and V. S. Hedna . 2013. “Neuroinflammation After Acute Ischemic Stroke: A Volcano Hard to Contain.” Chinese Journal of Contemporary Neurology and Neurosurgery 13, no. 11: 964–970.

[brb371623-bib-0022] Mohd Azmi, N. A. S. , N. Juliana , S. Azmani , et al. 2021. “Cortisol on Circadian Rhythm and Its Effect on Cardiovascular System.” International Journal of Environmental Research and Public Health 18, no. 2: 676. 10.3390/ijerph18020676. 33466883 PMC7830980

[brb371623-bib-0023] Poisson, S. N. , S. C. Johnston , and S. A. Josephson . 2010. “Urinary Tract Infections Complicating Stroke: Mechanisms, Consequences, and Possible Solutions.” Stroke; A Journal of Cerebral Circulation 41, no. 4: e180–e184. 10.1161/STROKEAHA.109.576413. 20167905

[brb371623-bib-0024] Rubingh, J. , A. van der Spek , E. Fliers , and A. Boelen . 2020. “The Role of Thyroid Hormone in the Innate and Adaptive Immune Response During Infection.” Comprehensive Physiology 10, no. 4: 1277–1287. 10.1002/j.2040-4603.2020.tb00144.x. 32969509

[brb371623-bib-0025] Seetge, J. , B. Cséke , Z. N. Karádi , E. Bosnyák , and L. Szapáry . 2025. “Shifting Outcomes: Superior Functional Recovery in Embolic Stroke of Undetermined Source Compared to Cardioembolic Stroke.” Neurology International 17, no. 3: 35. 10.3390/neurolint17030035. 40137456 PMC11945417

[brb371623-bib-0026] Tai, S. H. , L. C. Chao , S. Y. Huang , et al. 2023. “Nicotinamide Deteriorates Post‐Stroke Immunodepression Following Cerebral Ischemia–Reperfusion Injury in Mice.” Biomedicines 11, no. 8: 2145. 10.3390/biomedicines11082145. 37626642 PMC10452067

[brb371623-bib-0027] Taroza, S. , D. Rastenytė , A. Podlipskytė , H. Kazlauskas , and N. Mickuvienė . 2020. “Nonthyroidal Illness Syndrome in Ischaemic Stroke Patients Is Associated With Increased Mortality.” Experimental and Clinical Endocrinology & Diabetes 128, no. 12: 811–818. 10.1055/a-0915-2015. 31158897

[brb371623-bib-0028] von Elm, E. , D. G. Altman , M. Egger , S. J. Pocock , P. C. Gøtzsche , and J. P. Vandenbroucke . 2007. “Strengthening the Reporting of Observational Studies in Epidemiology (STROBE) Statement: Guidelines for Reporting Observational Studies.” BMJ (Clinical Research Ed) 335, no. 7624: 806–808. 10.1136/bmj.39335.541782.AD PMC2034723 17947786

[brb371623-bib-0029] Wang, C. , Q. Zhang , M. Ji , J. Mang , and Z. Xu . 2021. “Prognostic Value of the Neutrophil‐to‐Lymphocyte Ratio in Acute Ischemic Stroke Patients Treated With Intravenous Thrombolysis: A Systematic Review and Meta‐Analysis.” BMC Neurology 21, no. 1: 191. 10.1186/s12883-021-02222-8. 33975565 PMC8111766

[brb371623-bib-0030] Wang, R. , H. Li , C. Ling , et al. 2023. “A Novel Phenotype of B Cells Associated With Enhanced Phagocytic Capability and Chemotactic Function After Ischemic Stroke.” Neural Regeneration Research 18, no. 11: 2413–2423. 10.4103/1673-5374.371365. 37282471 PMC10360087

[brb371623-bib-0031] Westendorp, W. F. , C. Dames , P. J. Nederkoorn , and A. Meisel . 2022. “Immunodepression, Infections, and Functional Outcome in Ischemic Stroke.” Stroke; A Journal of Cerebral Circulation 53, no. 5: 1438–1448. 10.1161/STROKEAHA.122.038867. 35341322

[brb371623-bib-0032] Wu, K. , B. Jelfs , K. Neville , and Q. Fang . 2023. “Dynamic Brain Behaviours in Stroke: A Longitudinal Investigation Based on fMRI Analysis.” arXiv:2401.08607.

[brb371623-bib-0033] Yu, S. , J. Yan , R. Logan , et al. 2024. “Euthyroid Sick Syndrome Predicts the Risk of Ischemic Stroke‐Associated Pneumonia in the Acute Stage of Ischemic Stroke: A Nested Case‐Control Study.” Frontiers in Endocrinology 15: 1438700. 10.3389/fendo.2024.1438700. 39588332 PMC11586193

[brb371623-bib-0034] Zhang, S. , Z. Su , and X. Wen . 2024. “Association of T3/T4 Ratio With Inflammatory Indicators and All‐Cause Mortality in Stroke Survivors.” Frontiers in Endocrinology 15: 1509501. 10.3389/fendo.2024.1509501. 39845883 PMC11750665

[brb371623-bib-0035] Zhu, J. , X. Xia , Q. Xu , T. Feng , and A. Wang . 2025. “Use of the Modified Rankin Scale for Outcome Assessment and Statistical Methods in Stroke Clinical Trials.” Chinese Journal of Stroke 20, no. 8: 1029–1034.

